# Securing data management systems in perfusion: A narrative review on cybersecurity and clinical risk

**DOI:** 10.1177/02676591261417526

**Published:** 2026-01-12

**Authors:** Youssef El Dsouki, Ignazio Condello, Calogero Iacona, Samir El Daccache, Roberto Lorusso

**Affiliations:** 1 Faculty of Health, Medicine and Life Sciences, 82246 Maastricht University Medical Centre MUMC, Maastricht, The Netherlands; 2 School of Medicine and Surgery, 556391 University of Varese Insubria, University of Insubria, Varese, Italy; 3 Computer and Communication Sciences at ISSEA, Polytechnic University of Lugano, Lugano, Switzerland; 4 Department Complex System Engineering, 629587 University of Technology of Compiègne, Compiègne, France; 5 Cardiovascular Research Centre Maastricht CARIM, Maastricht, The Netherlands

**Keywords:** perfusion informatics, cardiopulmonary bypass, data management, cybersecurity, integrity, patient safety

## Abstract

**Background:**

The increasing digitization of perfusion practice has led to the integration of advanced data management systems (DMS) that record and analyze intraoperative variables in real time. While these systems improve accuracy, traceability, and clinical decision-making, they also expose perfusion workflows to significant cybersecurity risks. Given that perfusion data directly reflect artificial circulation, any loss of integrity or service can have immediate clinical consequences.

**Materials and Methods:**

A comprehensive narrative review was conducted to synthesize current knowledge on cybersecurity in perfusion informatics and performance. The research strategy combined systematic database searches (PubMed, Scopus, IEEE Xplore, and Web of Science) with targeted grey literature review (regulatory frameworks and institutional reports). Inclusion criteria focused on articles published between 2010 and 2024 addressing healthcare cybersecurity, perfusion informatics, and medical device security. Studies were analyzed qualitatively to identify recurring themes, technological approaches, and governance models. Reference cross-checking ensured comprehensive coverage, and terminology was standardized according to ISO/IEC 27001 principles. The review followed an integrative, non-meta-analytic design, emphasizing conceptual synthesis rather than statistical aggregation.

**Results:**

Thirty-three primary and secondary sources were identified. The literature reveals a consistent pattern of vulnerabilities across clinical DMS, with perfusion systems being particularly exposed to ransomware, data tampering, and service interruption. The review highlights the centrality of the Confidentiality–Integrity–Assurance of Service triad as a guiding model. Key strategies identified include encryption and role-based access control, blockchain audit trails, federated learning, and post-quantum cryptography. However, empirical studies focusing specifically on perfusion remain scarce, underscoring a major research gap.

**Conclusions:**

Cybersecurity in perfusion informatics must evolve from a technical adjunct to a clinical mandate. Integrating security frameworks into perfusion workflows supported by legal compliance (HIPAA, GDPR, ISO 27001) and ethical governance is essential to preserve patient safety. Future research should prioritize multicenter simulations, resilience testing, and cross-disciplinary training to ensure the continuity, integrity, and trustworthiness of digital perfusion systems.

## Introduction

The contemporary practice of perfusion stands at the intersection of medicine, engineering, and information technology.^1^ Over the last two decades, digital transformation and progress have profoundly reshaped how perfusionists monitor, document, analyze and store intraoperative data.^2^ Advanced data management systems (DMS) now integrate multiple physiologic streams including hemodynamic variables, gas exchange, indexed oxygen delivery (DO_2_i), and cerebral oximetry into a unified digital environment.^3^ These systems have redefined perfusion as not only a clinical discipline but also an informatics domain, where the quality and security of data directly influence patient outcomes.^4^ While digitalization has brought undeniable advantages, greater accuracy, automation, and medico-legal accountability, it has also exposed such systems to the cyber vulnerability of critical medical infrastructure. Cyber incidents in healthcare, such as ransomware or denial-of-service attacks, have demonstrated the fragility of interconnected systems.^5^ Within the perfusion setting, where real-time data continuity is essential to appropriate patient management and survival, any compromise in system integrity or service availability could have catastrophic intraoperative or perioperative consequences.^6^ Recent scientific work in secure clinical data infrastructures reinforces these concerns, emphasizing risk governance frameworks, Research Data Management compliance, and artificial intelligence applied to cybersecurity in clinical trials. These emerging trends confirm the urgency of integrating cybersecurity principles directly into perfusion workflows.^7^ Despite the exponential growth of literature on healthcare cybersecurity, perfusion remains an underexplored area. Research has predominantly focused on data-driven perfusion, automation, and clinical analytics, whereas systematic analyses of data protection, encryption protocols, or cyber-resilience strategies specific to perfusion environments are scarce.^7,8^ The few studies available emphasize the absence of standardized frameworks, limited cybersecurity awareness among clinical teams, and the urgent need for interdisciplinary collaboration between perfusionists, biomedical engineers, and information security experts.^8^ Risk stratification models specific to perfusion linking attack type, severity, and clinical consequence are virtually absent, representing a critical research gap. This narrative review aims to consolidate the fragmented body of knowledge regarding cybersecurity in perfusion DMS.^9,10^ By synthesizing findings from healthcare informatics, regulatory frameworks (HIPAA, GDPR, ISO 27001), and emerging technologies, such as blockchain, artificial intelligence, and post-quantum cryptography, the review seeks to (1) identify vulnerabilities specific to perfusion informatics, (2) outline effective mitigation strategies, and (3) advocate for a culture of digital resilience within perfusion practice, (4) propose direction for future empirical research and cyber-risk stratification models tailored to extracorporeal workflows. Ultimately, securing perfusion data means securing the patient’s physiology a responsibility that extends beyond technical compliance to the ethical core of patient safety (Figure 1).

**Figure 1.**
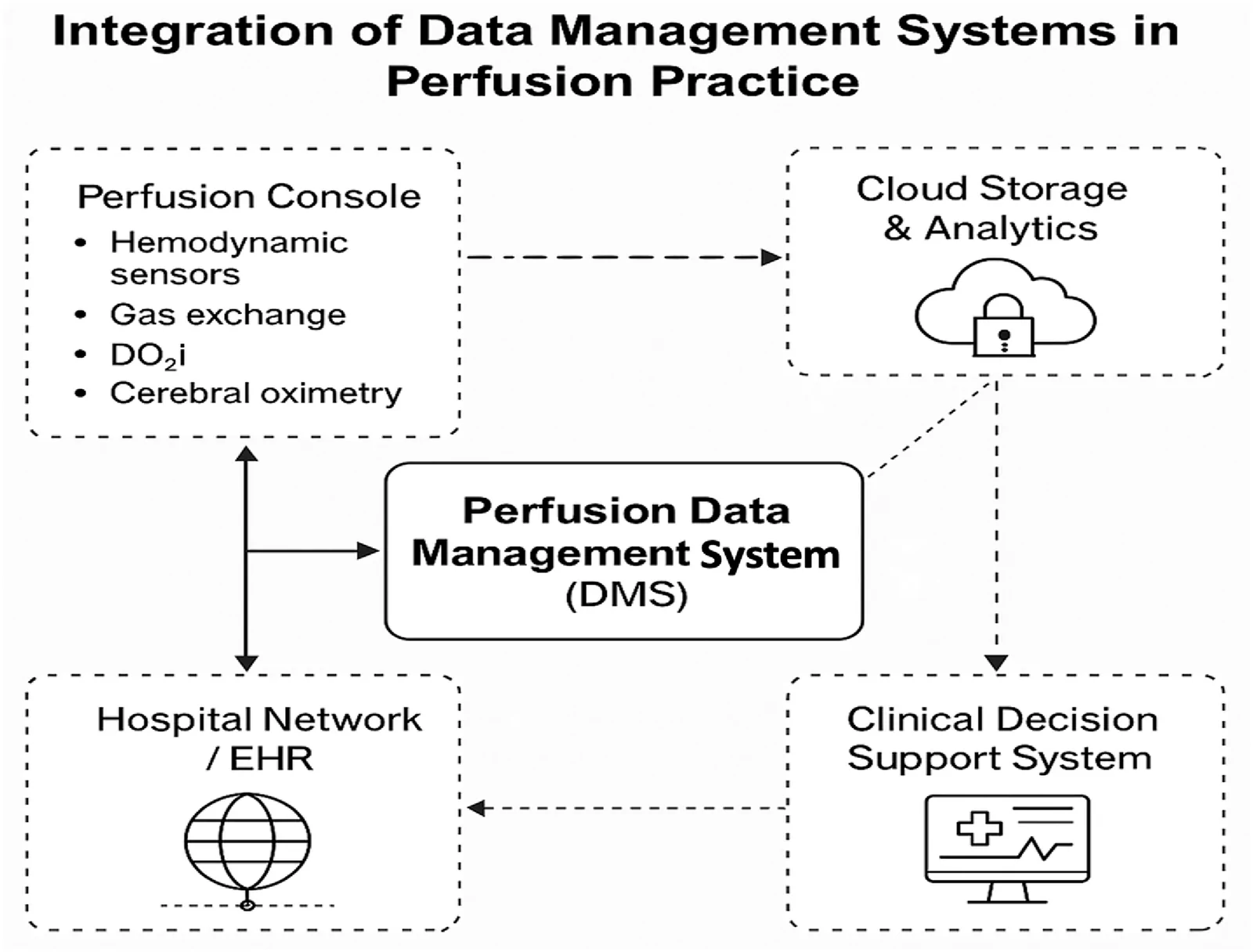
Integration of data management systems in perfusion practice. Schematic representation of the digital ecosystem surrounding perfusion data management systems (DMS). The diagram illustrates how perfusion consoles, hospital information networks, cloud analytics, and clinical decision support tools exchange data in real time. Arrows represent data flow, while dashed lines indicate network connections and potential cybersecurity interfaces.

## Materials and methods

This study was conducted as a narrative review with the objective of synthesizing contemporary evidence, conceptual frameworks, and expert perspectives on cybersecurity and data integrity within extracorporeal circulation and related perfusion management related to data management systems. The narrative review methodology was selected to enable a broad and integrative exploration of literature from multiple disciplines—clinical, technical, ethical, and regulatory reflecting the complex, cross-sector nature of digital transformation in perfusion. Unlike systematic reviews, this approach privileges conceptual synthesis and interpretive depth over quantitative aggregation, focusing on emerging patterns and relationships among diverse sources.

A comprehensive bibliographic search was conducted, using the databases PubMed/MEDLINE, Scopus, Web of Science, and IEEE Xplore. Additional searches were performed through Google Scholar, professional society repositories, and institutional archives to identify grey literature and relevant policy or technical documents. The search was complemented by an examination of international regulatory frameworks and standards governing healthcare data security, including the Health Insurance Portability and Accountability Act (HIPAA),^11^ the General Data Protection Regulation (GDPR),^12^ and the ISO/IEC 27001:2013 information security management system standard.^13^

The search strategy was structured around three conceptual domains aligned with the review’s analytical focus:

(1) *Perfusion and extracorporeal technology*, including terms such as “perfusion informatics,” “cardiopulmonary bypass,” and “extracorporeal circulation”;
(2) *Cybersecurity and data governance*, using terms such as “cybersecurity,” “information security,” “data integrity,” “healthcare IT,” and “confidentiality”;
(3) *Emerging technologies and ethical frameworks*, including “blockchain,” “federated learning,” “artificial intelligence,” “post-quantum cryptography,” and “digital ethics.”

The search was limited to English-language publications published between 2010 and 2024, a period corresponding to the rapid expansion of digital technologies and data integration in healthcare. Eligible materials included peer-reviewed journal articles, systematic and narrative reviews, editorials, and official standards or guidelines.^1,2,4–6^ Data integrity, confidentiality, and governance frameworks^7,8,11,12,13,14^ emerging technologies relevant to perfusion informatics, such as blockchain, federated learning, artificial intelligence, and post-quantum cryptography were also investigated.^3,5,9,10,15,16^ Publications not directly related to healthcare, non–peer-reviewed commentaries, and papers lacking methodological transparency were excluded.

All retrieved records were screened for relevance through a multi-stage review process. Titles and abstracts were first assessed to remove duplicates and unrelated content, followed by full-text evaluation of potentially eligible studies. Reference lists of included papers were examined to identify additional sources through citation chaining. Only references indexed in recognized databases (PubMed, Scopus, IEEE Xplore) and verified for bibliographic accuracy were retained for analysis. The collected data were analyzed through a qualitative, thematic synthesis, emphasizing interpretation and conceptual integration rather than quantitative metrics. The evidence was organized into four thematic domains that emerged across the literature: (1) cybersecurity threats and vulnerabilities in healthcare; (2) technological innovation and data-driven perfusion; (3) emerging paradigms and resilience strategies; and (4) ethical and regulatory foundations for data governance. The synthesis was guided by the Confidentiality Integrity Assurance of Service (CIAS) model, adapted from information security theory to the clinical domain of perfusion. This conceptual framework was used to interpret cybersecurity as not merely a technical safeguard, but a core component of patient safety and ethical responsibility. All included references were cross-checked for publication authenticity, methodological coherence, and thematic relevance. The overall process was conducted to ensure transparency, reproducibility, and scholarly rigor, consistent with current best practices for narrative reviews in biomedical literature.

## Results

A total of 48 records were initially identified through database searching, of which 16 articles met the inclusion criteria after full-text evaluation and verification of bibliographic authenticity. The reviewed literature revealed a multifaceted and interdisciplinary body of evidence describing the intersection between cybersecurity, healthcare data management, and perfusion informatics.

### Cybersecurity threats and vulnerabilities in healthcare data systems

The increasing digitalization of healthcare infrastructures has expanded both operational efficiency and exposure to cyber threats. Kruse et al.^1^ and Martin et al.^2^ demonstrated that healthcare environments remain highly vulnerable due to outdated infrastructures, heterogeneous device ecosystems, and insufficient personnel training. The prevalence of data breaches, ransomware, and phishing attacks has escalated globally, frequently exploiting human factors such as weak authentication or procedural negligence rather than purely technical flaws.^6^ Recent contributions have highlighted the sophistication of cyberattacks targeting clinical decision systems. Bortsova et al.^4^ showed that adversarial attacks can manipulate medical imaging algorithms, distorting diagnostic accuracy and potentially altering patient management. Likewise, Yeo and Banfield^6^ emphasized that human error, workflow pressures, and poor password management remain major causes of electronic health record (EHR) vulnerabilities. Within perfusion practice, where real-time monitoring and uninterrupted data continuity are essential, these findings underscore the need for both technological hardening and human-factor resilience.

### Technological innovation and data-driven perfusion

The progressive integration of artificial intelligence (AI) and automation has transformed perfusion monitoring and data interpretation, while simultaneously introducing new cybersecurity challenges. Topol^3^ described the rise of high-performance medicine, in which AI systems dynamically process clinical data to guide decision-making. Such systems demand stringent safeguards to preserve data integrity and prevent unauthorized manipulation or steal. Osama et al.^5^ explored the Internet of Medical Things (IoMT) and Healthcare 4.0 frameworks, which connect perfusion devices and extracorporeal systems within hospital networks. While this interconnectivity improves clinical coordination and data traceability, it also broadens the attack surface, exposing multiple network endpoints to potential intrusion. Similarly, Farias et al.^14^ identified vulnerabilities in remote patient monitoring systems related to encryption gaps and inconsistent access control. Collectively, these studies indicate that as perfusion becomes increasingly data-centric, the security of communication protocols and device authentication must become a priority in clinical technology governance.

### Emerging security paradigms and technological solutions

Several authors have proposed advanced digital architectures to enhance data security and clinical resilience. Taherdoost^15^ analyzed blockchain technology as a decentralized and tamper-proof ledger capable of securing medical data trails and ensuring accountability within perfusion data logs and ECMO registries. Teo et al.^9^ examined federated machine learning, a distributed AI approach that enables multi-institutional data sharing without compromising privacy a model particularly promising for collaborative perfusion research. Costa et al.^10^ addressed the implementation of post-quantum cryptography, proposing encryption models resistant to quantum computational attacks for long-term healthcare data protection. These technologies, though still in early stages of translation, align with the Confidentiality–Integrity–Assurance of Service (CIAS) model, reinforcing continuous service assurance even under cyber stress. Complementary frameworks, such as the Cloud Security Alliance (CSA) guidelines^16^ and ISO/IEC 27001:2013 standard,^13^ provide operational foundations for encryption, redundancy, and continuous vulnerability assessment within healthcare IT systems (Table 1).

**Table 1.** Summary of key findings from the narrative review.

Thematic domain	Key references	Main findings	Relevance to perfusion practice
1. Cybersecurity threats and vulnerabilities in healthcare data systems	Kruse et al.^1^; Martin et al.^2^; Bortsova et al.^4^; Yeo & Banfield^6^	Healthcare systems are increasingly exposed to ransomware, phishing, and adversarial attacks; vulnerabilities often arise from outdated systems and human error.	Highlights the need for robust cybersecurity protocols and staff training to prevent data loss or manipulation during extracorporeal procedures.
2. Technological innovation and data-driven perfusion	Topol^3^; Osama et al.^5^; Farias et al.^14^	Integration of AI, IoMT, and automation improves data-driven decision-making but introduces new risks in data transmission and device interoperability.	Demonstrates that digital perfusion systems require secure communication channels and authentication to ensure patient safety and data integrity.
3. Emerging security paradigms and technological solutions	Taherdoost^15^; Teo et al.^9^; Costa et al.^10^; CSA^16^; ISO 27001^13^	Blockchain, federated learning, and post-quantum cryptography represent promising models for resilient, privacy-preserving data infrastructures.	Provides a technological roadmap for secure perfusion data management and long-term protection of perfusion records.
4. Ethical and regulatory perspectives on digital governance	McKeown et al.^7^; Goodman^8^; Yeo & Banfield^6^; HIPAA^11^; GDPR^12^	Ethical consent, data reuse, and compliance with regulatory frameworks are essential for digital trust; most failures stem from inadequate compliance or awareness.	Emphasizes cybersecurity as a core component of ethical practice and patient safety within perfusion workflows.

### Ethical and regulatory perspectives on data governance

Beyond technological measures, cybersecurity in perfusion also encompasses ethical, legal, and governance dimensions. McKeown et al.^7^ and Goodman^8^ have underscored the ethical imperatives of informed consent, data reuse, and transparency in algorithmic systems, which form the basis of digital trust in healthcare. The HIPAA Security Rule^11^ and General Data Protection Regulation (GDPR)^12^ currently represent the cornerstone regulatory instruments ensuring confidentiality, integrity, and availability of sensitive patient information. Yeo and Banfield^6^ observed that security failures often stem not from regulatory absence but from inconsistent compliance and limited awareness among clinical personnel. This suggests the need for a culture of cybersecurity, in which perfusionists, clinicians, and biomedical engineers are trained to integrate ethical and digital risk considerations into daily clinical practice (Figure 2).

**Figure 2.**
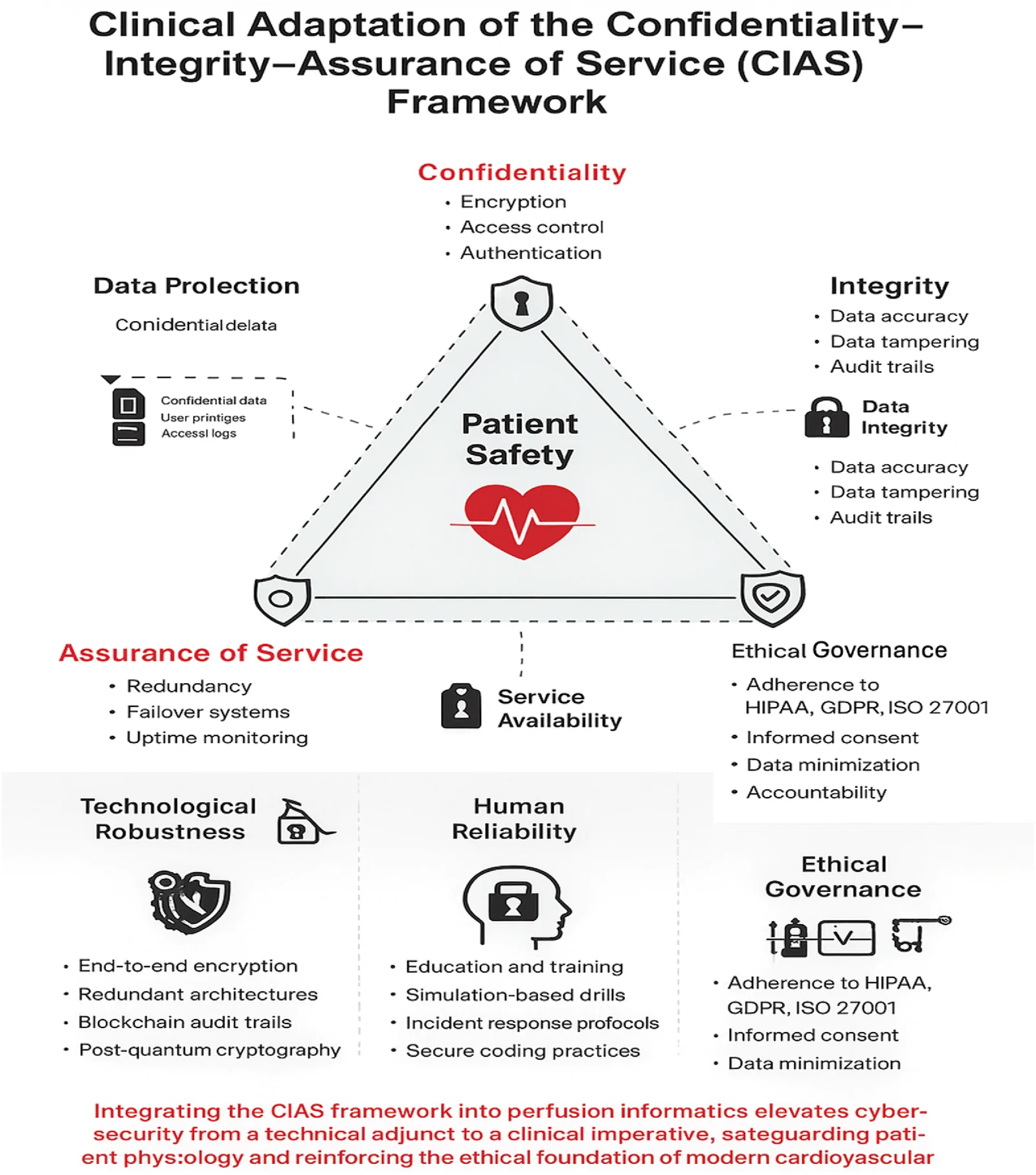
Clinical adaptation of the confidentiality integrity assurance of service (CIAS) framework. Conceptual diagram illustrating the adaptation of the confidentiality integrity assurance of service (CIAS) model to perfusion data management systems. Each pillar confidentiality, integrity, and assurance of service contributes to patient safety through technological robustness, human reliability, and ethical governance.

## Discussion

Perfusion DMS must therefore be recognized as safety-critical systems whose security demands the same rigor applied to life-support devices.^14^ In this context, the conceptual triad of confidentiality, integrity, and assurance of service provides a robust foundation for evaluating digital risk.^15^ Within perfusion, these constructs must be operationalized relative to physiological risk, time-to-impact, and system dependency, forming the basis for structured cyber-risk stratification. Across the studies analyzed, a clear convergence emerges: as perfusion systems become increasingly interconnected with hospital information networks and cloud-based infrastructures, their exposure to cyber threats expands proportionally. Kruse et al.^1^ and Martin et al.^2^ showed that healthcare institutions remain highly susceptible to ransomware, phishing, and data corruption attacks, largely due to outdated infrastructures and insufficient staff awareness. These findings are mirrored in the perfusion environment, where real-time monitoring and device interoperability are essential to patient survival. Perfusion-specific cyber-attack pathways illustrate potential clinical severity, including manipulation of real-time pump flow values, air bubble detector spoofing, oxygenator performance data corruption, network latency degrading DO_2_i calculations, and exploitation of cardiopulmonary bypass firmware vulnerabilities.^3,4^ These demonstrate that manipulated perfusion telemetry may produce physiological instability independent of operator skill.^5^ Furthermore, as highlighted by Yeo and Banfield,^6^ human factors including weak authentication practices and poor compliance with security protocols remain among the most common contributors to breaches, underscoring the necessity of integrating cybersecurity literacy into clinical education. Technological innovation has simultaneously driven progress and complexity. Artificial intelligence, automation, and the Internet of Medical Things (IoMT) have revolutionized data interpretation, predictive modeling, and system feedback in perfusion. Topol^3^ described the emergence of “high-performance medicine,” wherein continuous data processing supports real-time clinical judgment. However, as Osama et al.^5^ and Farias et al.^14^ observed, increased interconnectivity also amplifies systemic risk: the same digital pathways that enable precision monitoring can serve as entry points for cyber intrusion. In this sense, the perfusionist operates within an increasingly hybrid ecosystem part mechanical, part digital where safety depends on both physiological and informational control (Figure 3).

**Figure 3.**
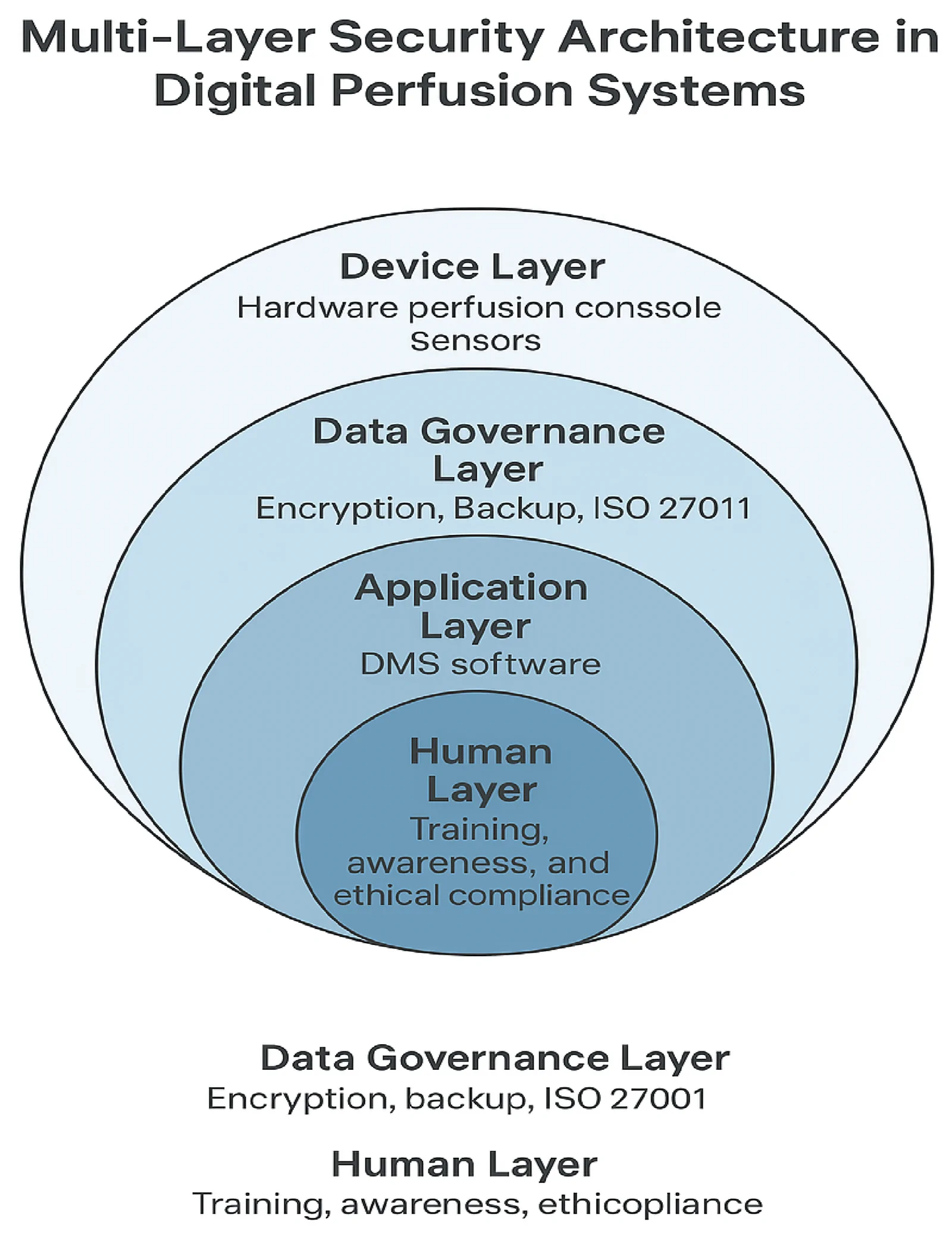
Multi-layer security architecture in digital perfusion systems. Schematic representation of the multi-layer cybersecurity structure in perfusion informatics. Each concentric layer from human to device illustrates a complementary level of protection integrating technical, procedural, and ethical safeguards to ensure data integrity and patient safety.

The secure integration of perfusion hardware and hospital software thus becomes a new dimension of patient safety, requiring not only robust technical defenses but also adaptive operational awareness. Emerging technologies offer promising yet still developing solutions. Blockchain architectures, as discussed by Taherdoost,^15^ introduce tamper-proof audit trails that can enhance data integrity and traceability in perfusion recordkeeping. Federated learning, examined by Teo et al.,^9^ allows distributed data analysis without exposing patient identifiers, facilitating multi-institutional research while maintaining confidentiality. Similarly, Costa et al.^10^ proposed post-quantum cryptography as a necessary step toward long-term protection of sensitive clinical data. Together, these innovations align with the Confidentiality Integrity Assurance of Service (CIAS) framework, emphasizing not only data protection but also service resilience in the face of potential disruption. While their application in perfusion remains largely theoretical, these models outline a technological roadmap for future implementation, one that balances performance with security and interoperability. The guidelines of the Cloud Security Alliance^16^ and the ISO/IEC 27001:2013 standard^13^ further support this vision by defining structured risk management and encryption practices applicable to medical IT systems. Beyond technology, the ethical and regulatory dimensions of cybersecurity are equally pivotal. McKeown et al.^7^ and Goodman^8^ highlighted the need for transparency, consent management, and algorithmic accountability in health data governance. The regulatory pillars established by the HIPAA Security Rule^11^ and the General Data Protection Regulation (GDPR)^12^ collectively define a framework for confidentiality, integrity, and availability of healthcare data. However, as Yeo and Banfield^6^ observed, compliance alone is insufficient when institutional culture and professional awareness are lacking. In perfusion, this translates into the ethical imperative that every operator understands the digital footprint of their practice: how data are captured, stored, transmitted, and protected. Cybersecurity, in this context, becomes not merely a technical safeguard but a reflection of professional ethics and patient-centered care. Ultimately, the synthesis of current evidence suggests that the resilience of perfusion data management systems depends on the interplay of three interdependent dimensions: technological robustness, human reliability and ethical governance.^10^ Technological robustness entails the implementation of secure architectures, continuous vulnerability assessment, and redundancy planning to ensure uninterrupted data flow during extracorporeal support. Human reliability involves the cultivation of a culture of cybersecurity awareness, supported by training and simulation to reduce the likelihood of human error. Ethical governance integrates these elements within a transparent, patient-oriented framework that aligns with both regulatory compliance and the moral obligation to protect human life. In this perspective, cybersecurity in perfusion should be redefined as a clinical competence rather than an ancillary technical domain. Integrating such technologies into perfusion environments requires institutional governance, standardized protocols, and validated security performance metrics. Cybersecurity responsibilities also extend to medico-legal accountability. Duty-to-report norms, incident disclosure policies, forensic logging, and escalation pathways define governance structures that ensure transparency and traceability when perfusion-related cyber incidents occur. Institutional risk committees and regulatory reporting bodies must integrate cyber-incident classification specific to life-support systems.

Ultimately, securing perfusion DMS requires alignment of three domains:

• technological robustness,
• human reliability and education, and
• ethical and institutional governance.

Without this alignment, digital transformation risks undermining rather than enhancing patient safety.

### Future research

Future work should transition from conceptual discussions toward empirical cyber-resilience evaluation in perfusion environments, prioritizing:

- multicenter high-fidelity simulations of cyberattacks during CPB/ECMO procedures measuring clinical latency, failover reliability, and time-to-harm thresholds;
- penetration testing and firmware vulnerability assessments of perfusion console network interfaces;
- standardized risk-stratification models assigning severity scores based on physiological dependency, data integrity criticality, and potential harm escalation timelines;
- quantitative evaluation of cybersecurity training programs for perfusion and OR personnel with controlled pre-/post assessment of detection and response metrics;
- prospective observational registries capturing cybersecurity events affecting perfusion workflows and system availability.

Such research will support scalable cybersecurity-by-design requirements for perfusion DMS, inform regulatory expectations, and guide institutional governance models.

## Conclusions

The digitalization of perfusion practice has greatly improved accuracy, traceability, and clinical decision-making, yet it has also exposed critical vulnerabilities in data management and patient safety from external sources. This review highlights that cybersecurity in perfusion informatics must evolve from a technical concern to a core clinical responsibility. Three pillars underpin this evolution: technological robustness, human reliability, and ethical governance. Robustness entails secure architectures using encryption, redundancy, and emerging tools such as blockchain and post-quantum cryptography. Human reliability depends on continuous education and awareness among perfusionists and biomedical staff. Ethical governance requires adherence to established frameworks HIPAA, GDPR, and ISO/IEC 27,001 ensuring that data protection aligns with patient safety and professional integrity. Future efforts should focus on empirical validation, including resilience testing and interdisciplinary collaboration to develop security protocols tailored to perfusion systems. Protecting perfusion data ultimately means protecting the patient’s physiology; cybersecurity must therefore be recognized as a fundamental clinical competence within modern perfusion practice. Future work must develop perfusion-specific risk models, empirical resilience testing protocols, and standardized reporting frameworks for cyber incidents affecting extracorporeal support. Securing perfusion data is inseparable from safeguarding patient physiology and therefore constitutes a core competency of modern perfusion practice.

## Data Availability

All data supporting the findings of this study are available from the corresponding author upon reasonable request.*

## References

[1] Kruse CS Frederick B Jacobson T , et al. Cybersecurity in healthcare: a systematic review of modern threats and trends. Technol Health Care 2017; 25(1): 1–10. 10.3233/THC-161263 27689562

[2] Martin G Martin P Hankin C , et al. Cybersecurity and healthcare: how safe are we? BMJ 2017; 358: j3179. 10.1136/bmj.j3179 28684400

[3] Topol EJ . High-performance medicine: the convergence of human and artificial intelligence. Nat Med 2019; 25(1): 44–56. 10.1038/s41591-018-0300-7 30617339

[4] Bortsova G González-Gonzalo C Wetstein SC , et al. Adversarial attack vulnerability of medical image analysis systems: unexplored factors. Med Image Anal 2021; 73: 102141. 10.1016/j.media.2021.102141 34246850

[5] Osama M Ateya AA Sayed MS , et al. Internet of medical things and healthcare 4.0: trends, requirements, challenges, and research directions. Sensors (Basel) 2023; 23(17): 7435. 10.3390/s23177435 PMC10490658 37687891

[6] Yeo LH Banfield J . Human factors in electronic health records cybersecurity breach: an exploratory analysis. Perspect Health Inf Manag 2022; 19(2): 1i. PMC9123525 35692854

[7] Cybersecurity GVS . Research data management (RDM), and regulatory compliance in clinical trials. Rev Recent Clin Trials 2025. Epub ahead of print. DOI: 10.2174/0115748871360236250901072442. 40947741

[8] Goodman KW . Ethics, information technology, and public health: new challenges for the COVID-19 era. J Law Med Ethics 2020; 48(1_suppl): 124–129. 10.1111/j.1748-720X.2010.00466.x 20446984

[9] Teo ZL Jin L Li S , et al. Federated machine learning in healthcare: a systematic review on clinical applications and technical architecture. Cell Rep Med 2024; 5(2): 101419. 10.1016/j.xcrm.2024.101419 PMC10897620 38340728

[10] Costa VLRD López J Ribeiro MV . A system-on-a-chip implementation of a post-quantum cryptography scheme for smart meter data communications. Sensors (Basel) 2022; 22(19): 7214. 10.3390/s22197214 PMC9572865 36236316

[11] U.S. Department of Health and Human Services . HIPAA security rule. HHS, 2013. https://www.hhs.gov/hipaa/for-professionals/security/laws-regulations/index.html

[12] European Union . General data protection regulation (GDPR). European Union; 2016.

[13] ISO/IEC 27001 . Information technology – security techniques – information security management systems. International Organization for Standardization, 2013.

[14] Farias FAC Dagostini CM Bicca YA , et al. Remote patient monitoring: a systematic review. Telemed J e Health 2020; 26(5): 576–583. 10.1089/tmj.2019.0066 31314689

[15] Taherdoost H . Privacy and security of blockchain in healthcare: applications, challenges, and future perspectives. Sci 2023; 5: 41.

[16] Cloud Security Alliance . Security guidance for critical areas of focus in healthcare cloud computing. CSA, 2021.

